# Apoptosis-associated speck-like protein containing a CARD regulates the growth of pancreatic ductal adenocarcinoma

**DOI:** 10.1038/s41598-021-01465-2

**Published:** 2021-11-16

**Authors:** Mitsuhito Koizumi, Takao Watanabe, Junya Masumoto, Kotaro Sunago, Yoshiki Imamura, Kozue Kanemitsu, Teru Kumagi, Yoichi Hiasa

**Affiliations:** 1 Department of Gastroenterology and Metabology, Ehime University Graduate School of Medicine, Ehime, Japan; 2 Department of Pathology, Ehime University Graduate School of Medicine and Proteo-Science Center, Ehime, Japan; 3 Post Graduate Medical Education Center, Ehime University Hospital, Ehime, Japan

**Keywords:** Cell growth, Gastroenterology

## Abstract

Apoptosis-associated speck-like protein containing a caspase recruitment domain (ASC) is a key adaptor protein of inflammasomes and a proapoptotic molecule; however, its roles in signal transduction in pancreatic ductal adenocarcinoma (PDAC) cells remain unknown. Here, we clarified the role and mechanisms of action of ASC in PDAC using clinical evidence and in vitro data. ASC expression in PDAC tissues was analyzed using public tumor datasets and immunohistochemistry results of patients who underwent surgery, and PDAC prognosis was investigated using the Kaplan–Meier Plotter. *ASC* expression in PDAC cells was downregulated using small-interfering RNA, and gene expression was assessed by RNA sequencing. Review of the Oncomine database and immunostaining of surgically removed tissues revealed elevated ASC expression in PDAC tumors relative to non-tumor tissue, indicating poor prognosis. We observed high *ASC* expression in multiple PDAC cells, with *ASC* silencing subsequently inhibiting PDAC cell growth and altering the expression of cell cycle-related genes. Specifically, *ASC* silencing reduced cyclin D1 levels and stopped the cell cycle at the G1 phase but did not modulate the expression of any apoptosis-related molecules. These results show that ASC inhibited tumor progression via cell cycle modulation in PDAC cells and could be a potential therapeutic target.

## Introduction

Pancreatic ductal adenocarcinoma (PDAC) is a very aggressive disease with the poorest prognosis among human cancers^1^. Despite the introduction of new treatment modalities in recent years, treatment options remain limited, and chemotherapy has not been fully effective^2,3^. Thus, there is an urgent need to identify novel targets and mechanisms underlying PDAC progression.

Apoptosis-associated speck-like protein containing a caspase recruitment domain [ASC; also called PYD- and CARD-domain-containing protein (PYCARD) and target of methylation-induced silencing 1 (TMS1)] reportedly forms aggregates in human myelocytic leukemia HL-60 cells undergoing apoptosis^4^. ASC is a key adaptor protein in the formation of various inflammasomes and plays crucial roles in caspase-1 activation and the secretion of interleukin (IL)-1β and IL-18 in innate immune cells^5–7^. However, ASC has also been identified as a target of TMS1 and a gene that is silenced by DNA methyltransferase 1 in breast cancer. Similar to that in breast cancer, *ASC* expression is decreased by methylation in cancers, such as melanoma^8^, ovarian cancer^9^, prostate cancer^10^, colorectal cancer^11^, and hepatocellular carcinoma^12^. It is also reportedly involved in the progression of melanoma^13^ and oral cavity squamous cell carcinoma^14^. However, the roles of ASC in PDAC have not been elucidated to date. These conflicting data suggest that the role of ASC in cancer might vary depending on the pathological tumor type, stage of tumor development, and tumor microenvironment^15^. In this study, we identified the role and mechanisms of ASC in PDAC based on clinical evidence and in vitro data obtained using PDAC cells.

## Methods

### Analysis of Oncomine data

To determine the expression pattern of ASC in PDAC, we used datasets from the Oncomine database (www.oncomine.org). The *ASC* gene was queried in the database, and results were filtered by selecting PDAC versus Normal Analysis. Data were displayed using Box Chart. P-values for each group were calculated using the Student’s *t* test. Details of standardized normalization techniques and statistical calculations are provided in the Oncomine website.

### Patients and pancreas specimens

PDAC specimens and adjacent normal pancreatic tissues were obtained from patients who underwent surgery at the Ehime University Hospital. All the specimens used in this study were obtained from eight samples collected between 2018 and 2019 from PDAC patients. Written informed consent was obtained from all enrolled participants, and the study protocol conformed to the ethical guidelines of the Declaration of Helsinki and was approved by the Institutional Review Board of Ehime University Hospital (approval no. 1712007).

### Immunohistochemistry (IHC) of PDAC specimens

Pancreatic tissues were fixed in formalin, sections (3-µm thick) were cut from each block, and adjacent sections were stained using IHC. Paraffin-embedded samples were dewaxed and rehydrated, and antigen retrieval was performed by autoclaving for 1 min at 110 °C in citrate buffer (pH 6.0). Endogenous peroxidase activity was inactivated by incubation with methanol containing 1% hydrogen peroxidase for 20 min. The sections were then incubated in 1% blocking goat serum for 30 min to reduce nonspecific reactions. For IHC, the sections were probed overnight with the anti-ASC antibody (1:200; 10500-1-AP; Proteintech, Tokyo, Japan) at 4 °C. The tissue sections were then incubated with a peroxidase-labeled secondary antibody (Histofine Simplestain Max POR; Nichirei, Tokyo, Japan) for 1 h at room temperature and then with Simple Stain DAB solution (Nichirei). Photomicrographs were obtained using a Nikon ECLPSE 50i with a Nikon digital camera (DS-F11; Nikon, Tokyo, Japan). Staining intensity was scored with no prior knowledge of clinicopathological results, in the following manner: 0, no staining; 1, weak staining; 2, moderate staining; and 3, strong staining. Immune-reaction intensity (optical density, OD) was examined using ImageJ (NIH, Bethesda, MD, USA) and calculated as OD = log [max intensity/mean intensity (max intensity = 255].

### The Kaplan–Meier Plotter

The Kaplan–Meier Plotter (https://kmplot.com/analysis/) is a web server for the discovery and validation of survival biomarkers^16^. We used the survival-analysis tool in the Kaplan–Meier Plotter to analyze the prognostic significance of *ASC* mRNA. Survival analysis was performed using the Kaplan–Meier method, and a hazard ratio (HR) with 95% confidence intervals (CIs) and a log-rank P-value were calculated. Kaplan–Meier Plotter was also used to compare the predictive value of several gene combinations in patients with low and high mutational burden.

### Cancer Cell Line Encyclopedia (CCLE) analysis

*ASC* mRNA levels in a series of cancers were analyzed using the CCLE (https://portals.broadinstitute.org/ccle/home). According to the website, raw microarray data of PDAC cell lines were converted to a single value for each probeset using the Robust Multi-array Average algorithm and quantile normalization.

### Cells and culture conditions

Four PDAC cell lines (PANC-1, BxPC-3, AsPC-1, and MIA PaCa-2), which were initially generated using samples from PDAC patients, and hTERT-HPNE cells were obtained from the American Type Culture Collection (Manassas, VA, USA). PANC-1 and MIA PaCa-2 cells were cultured in Dulbecco's modified Eagle medium (DMEM; Life Technologies, Carlsbad, CA, USA) supplemented with 10% fetal bovine serum (FBS; Life Technologies) and 1% penicillin. BxPC-3 and AsPC-1 cells were cultured in Roswell Park Memorial Institute (RPMI) 1640 medium supplemented with 10% FBS and 1% penicillin. hTERT-HPNE cells were cultured in 75% DMEM and 25% Medium M3 Base with 5% FBS, 10 ng/mL human recombinant epidermal growth factor, 5.5 mM d-glucose, and 750 ng/mL puromycin. Microphotographs were obtained after various treatments using an inverted microscope (AxioVertA1 FL; ZEISS, Oberkochen, Germany) equipped with an Axiocam MRm camera (ZEISS).

### RNA extraction, cDNA synthesis, and real-time reverse transcription polymerase chain reaction (RT-PCR)

Total RNA was extracted using TRIzol reagent (Thermo Fisher Scientific, Waltham, MA, USA), and the RNA was reverse transcribed using RT-PCR kits (Applied Biosystems, Tokyo, Japan). Real-time PCR was performed using a LightCycler 480 (Roche, Tokyo, Japan) with SYBR Green I (Roche). Sequences of the primers used to amplify human genes are indicated in Table S1. *Glyceraldehyde 3-phosphate dehydrogenase* served as an internal reference gene, and the relative change was calculated by relative quantification using the formula 2^−ΔΔCt^.

### Western blotting

Radioimmunoprecipitation assay buffer [50 mM HEPES, KOH (pH 7.9), 150 mM NaCl, 1.5 mM MgCl_2_, and 1.0% (v/v) NP-40] was used to lyse cells or homogenize tumors for protein extraction. For western blotting, 20 µg of protein was loaded onto 4–12% Bis–Tris gels (Life Technologies), followed by transfer onto Immobilon-P membranes (Millipore, Bedford, MA, USA) and incubation with the relevant primary antibodies (1:1000–3000 dilutions) (Table S2). Appropriate species-specific conjugated secondary antibody kits were obtained commercially (GE Healthcare, Tokyo, Japan). Proteins were detected using the ECL prime kit or the ECL kit (GE Healthcare) and an ImageQuant LAS 4000 system (GE Healthcare).

### RNA interference

Small-interfering (si)RNAs targeting ASC included the following: (1) 5′-AACUGGACCUGCAAGGACUUG-3′ (Sigma-Aldrich Japan, Tokyo, Japan)^17^ and (2) 5′-CCGCCGAGGAGCUCAAGAA-3′ (Hs_PYCARD_8145; Mission siRNAs; Sigma-Aldrich Japan). Control siGENOME non-targeting siRNA sequences (Thermo Scientific) included the following: 5′-UAAGGCUAUGAAGAGAUAC-3′, 5′-AUGUAUUGGCCUGUAUUAG-3′, 5′-AUGAACGUGAAUUGCUCAA-3′, and 5′-UGGUUUACAUGUCGACUAA-3′. Cell lines were transfected with 25 pM siRNA using Lipofectamine 2000 (Life Technologies), and 3 days after transfecting siRNA, mRNA and protein were extracted and analyzed.

### Cell-proliferation assay

Cell viability was quantified by an MTS assay (Promega, Fitchburg, WI, USA). Cells (2 × 10^3^) were seeded in 96-well plates and transfected with siRNA 1 day later (siRNA-transfection day was set as day 0). At each time point, cells were treated with MTS reagent and incubated for 1 h. Absorbance at 452 nm was recorded using a plate reader (Biochrom Asys Expert 96; Biochrom, Cambridge, UK).

### RNA sequencing (RNA-seq) and data analyses

Total RNA was collected from PANC-1 and AsPC-1 cells at 72 h after transfection with either control siRNA, *ASC* siRNA (1), or *ASC* siRNA (2), and the integrity of isolated RNA was verified using an Agilent 2100 Bioanalyzer (Agilent Technologies, Santa Clara, CA, USA). RNA samples with an RNA integrity number greater than eight were normalized to 100 ng/μL before further analyses. RNA-seq libraries were prepared using the Illumina TruSeq Stranded mRNA sample prep kit setA according to manufacturer instructions (Illumina, San Diego, CA, USA) and subsequently validated to ensure an average size of ~ 330 to ~ 340 bp using a 2100 Bioanalyzer and the Agilent DNA1000 kit. Paired-end reads (75 bp) were sequenced using the MiSeq reagent kit V3 150 cycle on a MiSeq system (Illumina). Mapping to human genome data hg19 was performed using Tophat (https://ccb.jhu.edu/software/tophat/index.shtml), and the expression analysis tool Cufflinks (http://cole-trapnell-lab.github.io/cufflinks/) was used to normalize each sample before determining the differences in expression between control siRNA (n = 3), *ASC* siRNA (1) (n = 3), and *ASC* siRNA (2) (n = 3) using the tmm package in R (https://www.r-project.org/). Raw and processed data were submitted to the Gene Expression Omnibus (GSE168842) database. Pathway and process enrichment analyses were performed using all genes that after alignment and normalization were found to be downregulated with an adjusted p < 0.05 using Metascape^18^ and gene set enrichment analysis (GSEA)^19^.

### Cell cycle analysis

Cells were seeded at a density of 1 × 10^6^ cells/well in 10-cm dishes. At 72 h after siRNA transfection, a BD Cycletest TM Plus DNA kit (BD Biosciences, Franklin Lakes, NJ, USA) was used to purify the sample according to manufacturer instructions, and cell cycle distribution was evaluated by flow cytometry (Gallios flow cytometer; Beckman Coulter, Brea, CA, USA).

### Apoptosis assay

PANC-1 cells were transfected with control siRNA or *ASC* siRNA, and a Mebstain apoptosis terminal deoxynucleotidyl transferase dUTP nick-end labeling (TUNEL) kit II (MBL, Nagoya, Japan) was used to detect oligonucleosomal DNA breaks in PANC-1 cells 3 and 5 days after transfection.

Proteome profiling was performed using commercially available human apoptosis proteome profiler arrays (R&D Systems, Minneapolis, MN, USA) according to manufacturer instructions. Expression levels of 35 apoptosis-related proteins (effector and signaling molecules) were evaluated by densitometric analyses of the arrays using ImageJ (NIH).

### Cytokine quantification in tumor cells

Tumor cells were plated onto 6-well plates in 2 mL DMEM or RPMI 1640 supplemented with 10% FBS and incubated for 24 h at 37 °C. Medium was replaced with 2 mL of fresh medium in each well, and the cells incubated for another 48 h. Supernatants were collected, and the levels of IL-1β were assessed using enzyme-linked immunosorbent assay (ELISA; human IL-1β/IL-1F2 Quantikine ELISA Kit; R&D Systems).

### Statistical analysis

All statistical analyses were performed using JMP software (v.14.0; SAS Institute, Tokyo, Japan). Data are expressed as the mean and standard deviation. Differences were analyzed using Student’s t-test, the Wilcoxon test, and χ^2^ test. Statistical significance was defined as p < 0.05 based on a two-tailed test. Correlations between two variables were evaluated by using Pearson's correlation coefficient.

## Results

### ASC is highly expressed in PDAC

**Figure 1:**
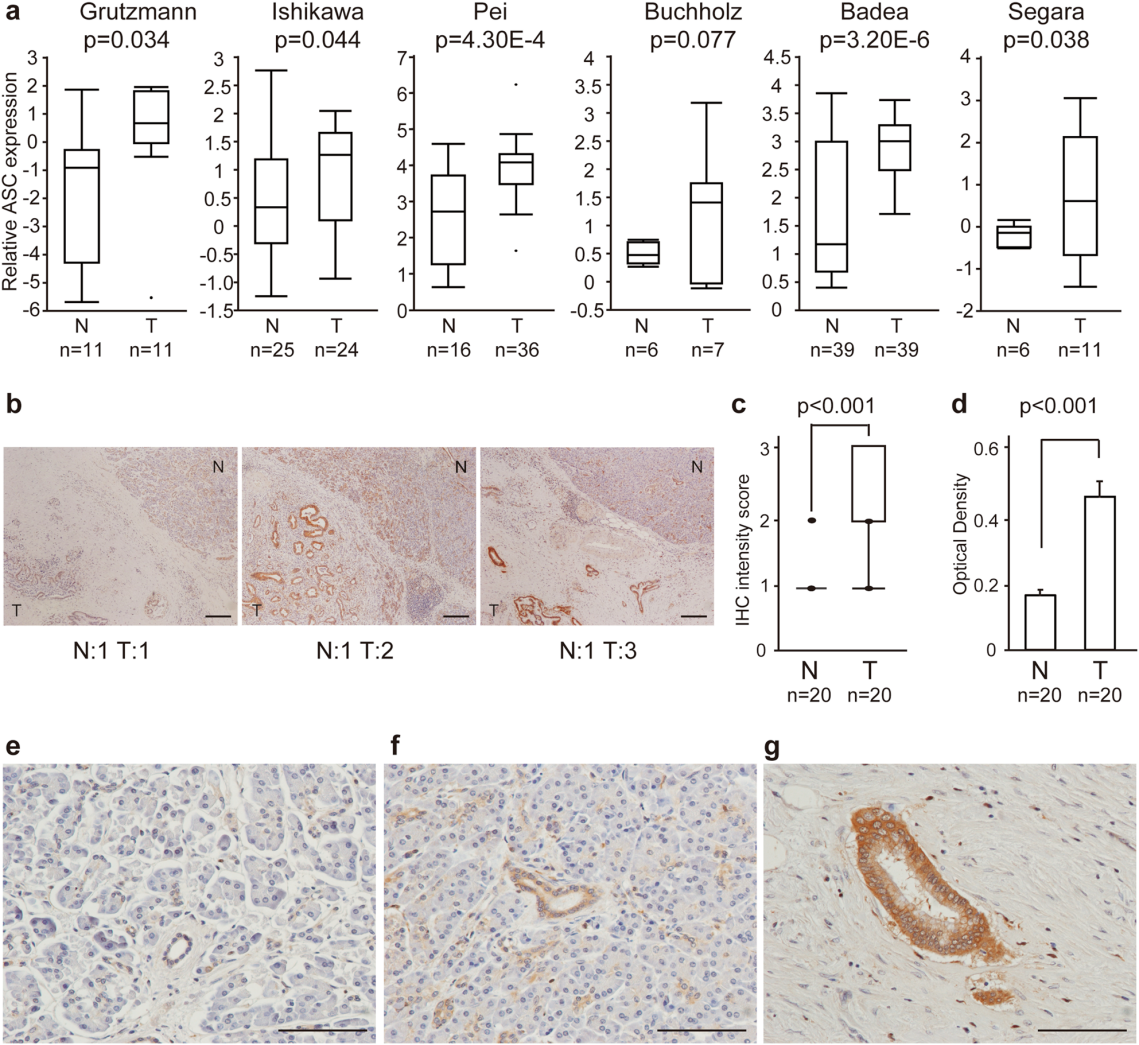
ASC expression according to Oncomine and clinical PDAC tissues. (**a**) Relative expression of *ASC* in normal/benign pancreas tissue (N) versus PDAC (T) according to six independent profiling datasets (surveyed using Oncomine). (**b**) Evaluation of ASC expression in human PDAC tissue according to IHC analysis. Data were generated from three representative PDAC tissues expressing various ASC levels and determined using the anti-ASC antibody. Scale bar: 200 μm. (**c**) IHC intensity score among 20 evaluated patients. (**d**) Immune-reaction intensity according to the OD and determined using ImageJ (n = 20). Error bars indicate the standard error of the mean based on eight independent experiments. (**e**–**g**) Expression of ASC protein in pancreatic tissue distant form the tumor (**e**), tissue surrounding the PDAC tissue (**f**), and PDAC tissue (**g**) according to IHC analysis. Scale bar: 100 μm.

Examination of the Oncomine database showed that *ASC* mRNA was significantly upregulated in PDAC in multiple microarray datasets^20–25^ (Fig. 1a). A microarray meta-analysis of these six datasets revealed that *ASC* mRNA was significantly upregulated in PDAC (p = 0.039). Accordingly, examination of immunostaining scores for the surgically removed tissues revealed that the immunostaining intensity was predominantly stronger in PDAC tissues relative to that in tissues surrounding the tumor (Fig. 1b,c). Quantitative assessment of the immunostaining results indicated a significantly higher OD in PDAC tissue relative to that in tissues surrounding the tumor (OD: 0.409 vs. 0.216, respectively; p = 0.0181) (Fig. 1d). Furthermore, ASC expression in ducts and acinar cells was weak in tissues distant from the tumor (Fig. 1e); however, we observed strong expression of ASC in either metaplastic or normal ducts within their nearby normal tissue relative to that in acinar tissue (Fig. 1f), and ASC expression was even stronger in cancerous lesions than surrounding tissues (Fig. 1g).

### High ASC expression predicts poor prognosis of PDAC

**Figure 2:**
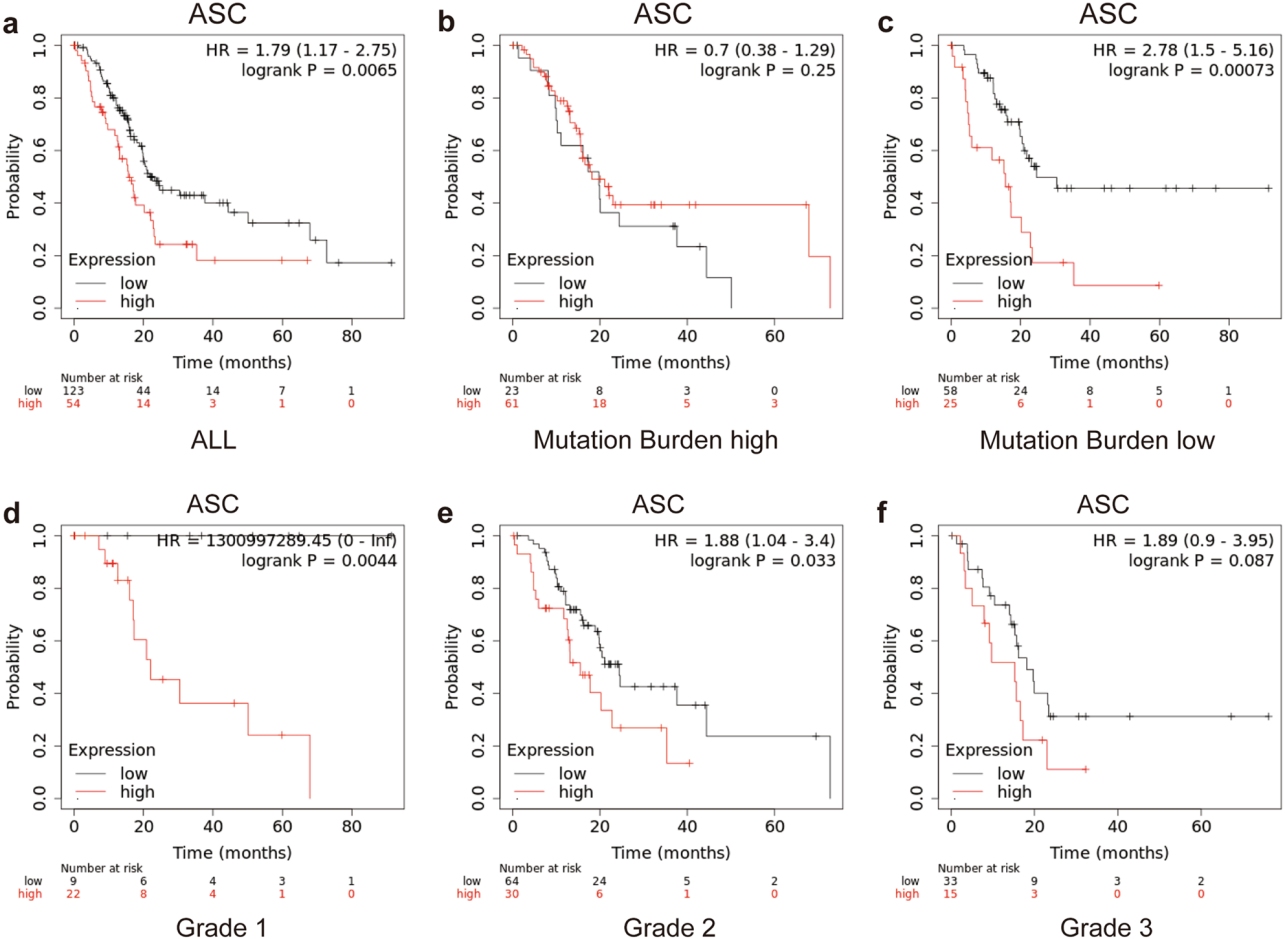
OS evaluated between PDAC patients with high and low expression of ASC. (**a**) OS of all PDAC patients (n = 177) and those with (**b**) high TMB (n = 84) and (**c**) low TMB (n = 83). (**d**) OS of PDAC patients with histological grade 1 (n = 31), (**e**) grade 2 (n = 94), and (**f**) grade 3 (n = 48) tumors. *TMB* tumor mutation burden.

To explore the clinical significance of ASC in PDAC, we performed Kaplan–Meier analysis, with the results showing that patients with elevated *ASC* mRNA expression had shorter overall survival (OS; n = 177; HR = 1.79, 95% CI 1.17–2.75) (Fig. 2a). We then determined whether *ASC* mRNA expression is predictive of outcomes in a cohort of tumors with low and high mutation burden. We found that elevated *ASC* mRNA expression was predictive of poor prognosis in a cohort of patients harboring tumors with low mutation burden (n = 83; HR = 2.78, 95% CI 1.5–5.16; log-rank p = 0.00073) (Fig. 2b), whereas high *ASC* mRNA expression did not contribute to prognosis in tumors with high mutation burden (n = 84; HR = 0.7, 95% CI 0.38–1.29; log-rank p = 0.25) (Fig. 2c). Based on evaluations of tumor grade, we found that elevated *ASC* mRNA expression was predictive of poor prognosis in a cohort of patients with grade 1 and grade 2 tumors but not grade 3 (grade 1: n = 31; HR = 1,300,997,289; 95% CI 0–inf; log-rank p = 0.0044; grade 2: n = 94; HR = 1.88, 95% CI 1.04–3.4; log-rank p = 0.033; and grade 3: n = 48; HR = 1.89, 95% CI 0.9–3.95; log-rank p = 0.087) (Fig. 2d–f).

### Downregulation of ASC expression inhibits the growth of PDAC cells

**Figure 3:**
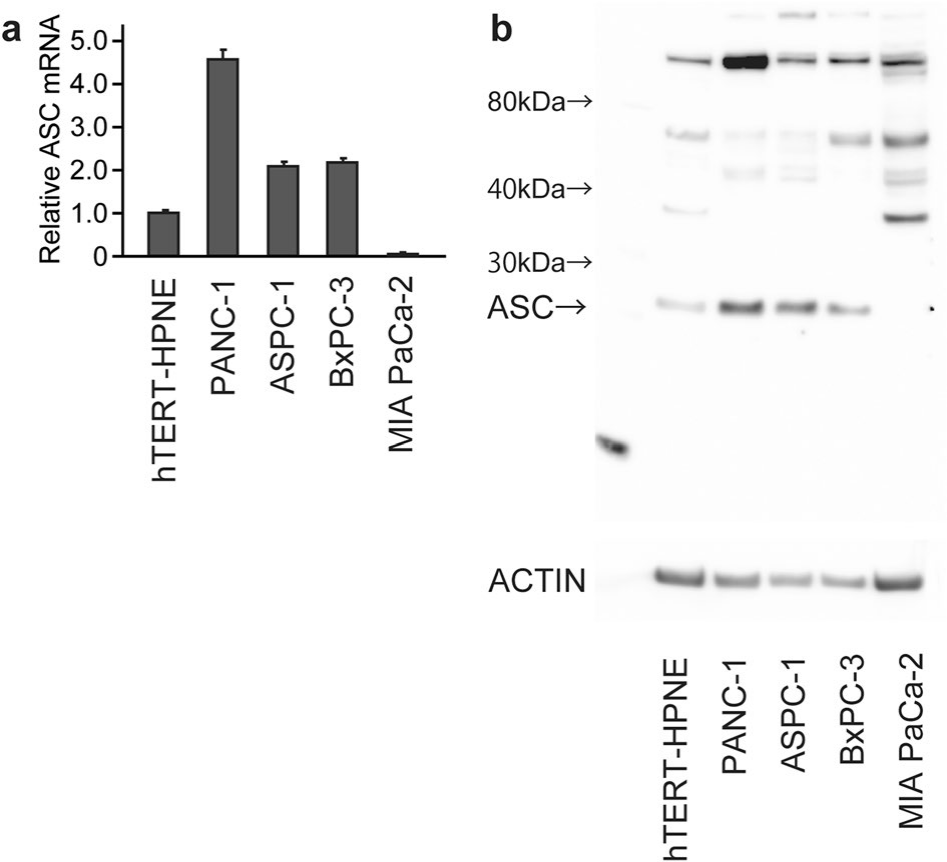
ASC expression in human PDAC cells. Four human PDAC cell lines and one human pancreatic duct cell line were used to screen levels of *ASC* mRNA and protein expression using quantitative real-time RT-PCR and western blot. (**a**) *ASC* mRNA expression. Data represent the mean ± standard error of the mean of four replicates. (**b**) ASC protein expression. The original gel images of Western blotting are shown in Fig. S6.

**Figure 4:**
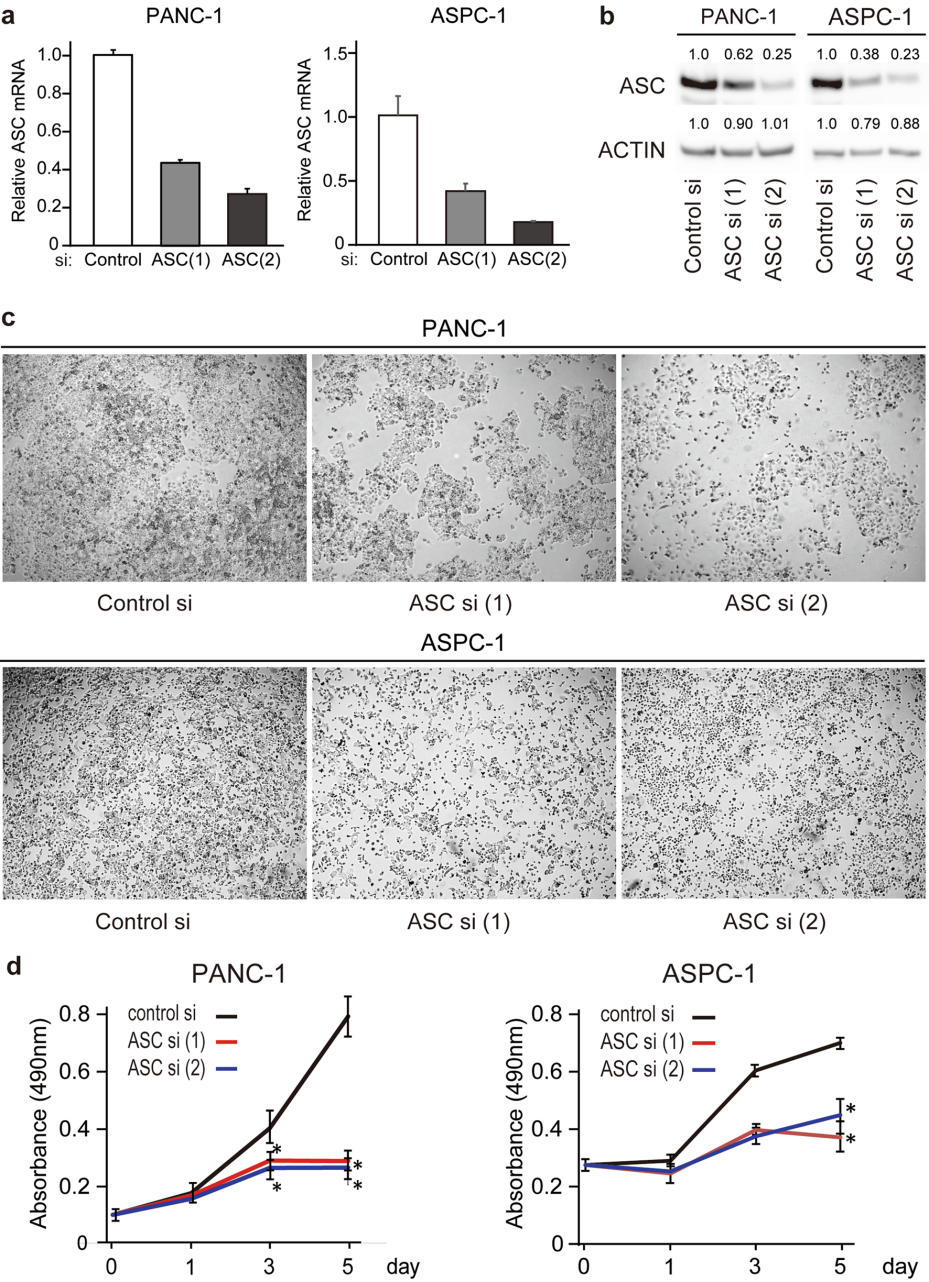
Changes in PDAC cells following *ASC* silencing. (**a**) *ASC* mRNA expression following transfection with control and *ASC* siRNA. Data represent the mean ± standard error of the mean of three to four replicates. (**b**) Western blot analysis of ASC protein expression following transfection with control and *ASC* siRNA. Relative densitometry data as compared with controls are given for each lane. The original gel images of Western blotting are shown in Fig. S7. (**c**) Photographs of wells on day 5 after siRNA transfection. (**d**) Viability of cells transfected with control or *ASC* siRNA. Data represent the mean ± standard error of the mean of three replicates. *p < 0.05, vs. control, Student’s *t* test.

We then examined *ASC* expression in PDAC cell lines, finding increased expression in PANC-1, ASPC-1, and BXPC-3 cells and decreased expression in Mia-PaCa-2 cells relative to that in the pancreatic ductal epithelial cell line hTERT-HPNE, which exhibits an acinar associated with the ductal metaplasia phenotype, according to real-time RT-PCR (Fig. 3a). Additionally, we confirmed *ASC* expression in PDAC cell lines using the CCLE database (Fig. S1). Additionally, western blotting identified high ASC expression in PANC-1, ASPC-1, and BxPC-3 cells, low expression in hTERT-HPNE cells, and no expression in MIA PaCa-2 cells (Fig. 3b). These findings are in agreement with those of our evaluation of human tissues, with high ASC expression observed in multiple cancer cell lines relative to normal pancreatic duct epithelial cells. To investigate the role of ASC, we knocked down *ASC* expression using two *ASC* siRNAs in PANC-1 and ASPC-1 cells, both of which exhibit relatively high *ASC* expression. Following confirmation of successful knockdown of ASC levels by real-time RT-PCR and western blotting (Fig. 4a,b), microscopy-based observation of cells revealed inhibited proliferation (Fig. 4c). Evaluation of cell proliferation by MTS assay confirmed this observation, and that this effect increased over time (p < 0.05) (Fig. 4d). Notably, suppressed proliferation following ASC knockdown was not observed in Mia PACA-2 or hTERT-HPNE cells (Fig. S2). These results indicated that ASC is mainly involved in PDAC cell proliferation.

### ASC regulates the cell cycle in PDAC cells

**Figure 5:**
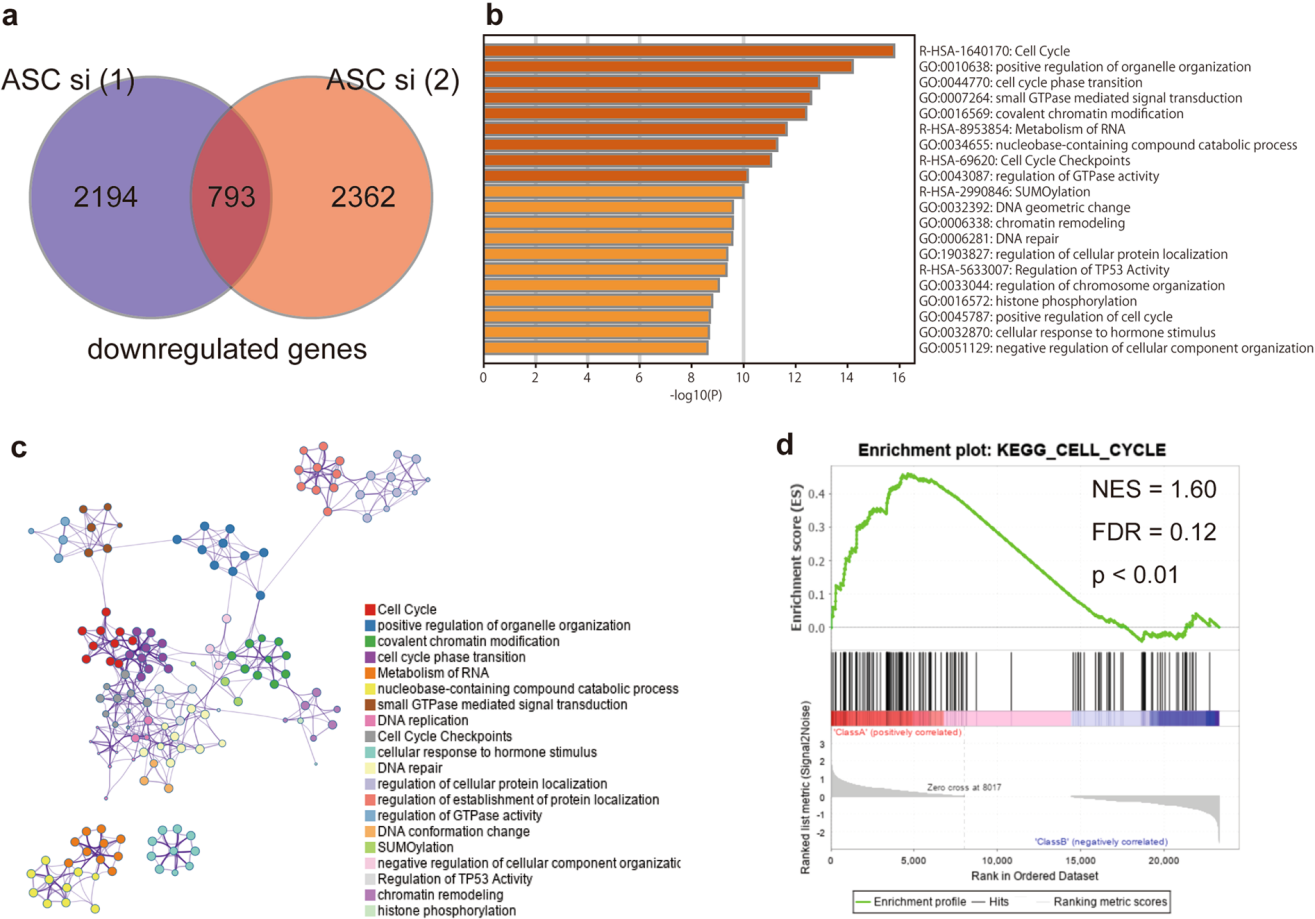
Functional enrichment analysis using Metascape and GSEA. (**a**) Venn diagram showing overlapping RNAs detected via RNA-seq analysis of downregulated RNAs at 72-h post-transfection of either *ASC* (1) or (2) siRNA and relative to control siRNA-transfected PANC-1 cells. (**b**) Functional enrichment results. Heatmap showing the top 20 clusters colored according to the p-value (darker color indicates a lower p-value). (**c**) Network of the top 20 clusters of enriched terms. Each node indicates an enrichment term colored by cluster ID. Nodes sharing the same cluster are generally located in close proximity. Terms with a kappa similarity ≥ 0.3 are connected. (**d**) GSEA for the gene signature of KEGG_CELL_CYCLE. *NES* normalized enrichment score, *FDR* false discovery rate.

To elucidate the detailed molecular mechanism underlying ASC-mediated PDAC cell proliferation, we performed RNA-seq analysis on control PANC-1 cells and *ASC-*silenced PANC-1 cells. We found that the total number of RNAs in the silenced cells significantly decreased relative to the control [*ASC* siRNA (1) and (2): 2194 and 2364, respectively]. Additionally, we analyzed 793 RNAs that were commonly reduced in the two *ASC* siRNA groups using Metascape (Fig. 5a). The results showed that significantly enriched signaling pathways included the cell cycle, positive regulation of organelle organization, and cell cycle phase transition. The top 20 clusters of significantly enriched terms are shown in Fig. 5b,c. Furthermore, GSEA revealed significant relationships between the expression of cell cycle-related genes (normalized enrichment score = 1.60; and false discovery rate = 0.12; p < 0.01). (Fig. 5d). Similar data were obtained in RNA-seq experiments using AsPC-1 cells (Fig. S3). Moreover, we observed no changes in the expression of apoptosis-related genes in either the PANC-1 or the AsPC-1 cell lines [Kyoto Encyclopedia of Genes and Genomes (KEGG) apoptosis pathway^26^: p = 0.72, q = 0.85 in PANC-1 cells; and p = 0.42, q = 0.72 in AsPC-1 cells]. These results indicated that the reduced cell-proliferation phenotypes observed in *ASC*-silenced cells were induced by altered expression of cell cycle-related genes.

### ASC knockdown reduces cyclin D1 (CCND1) level and stops the cell cycle at the G1 phase

**Figure 6:**
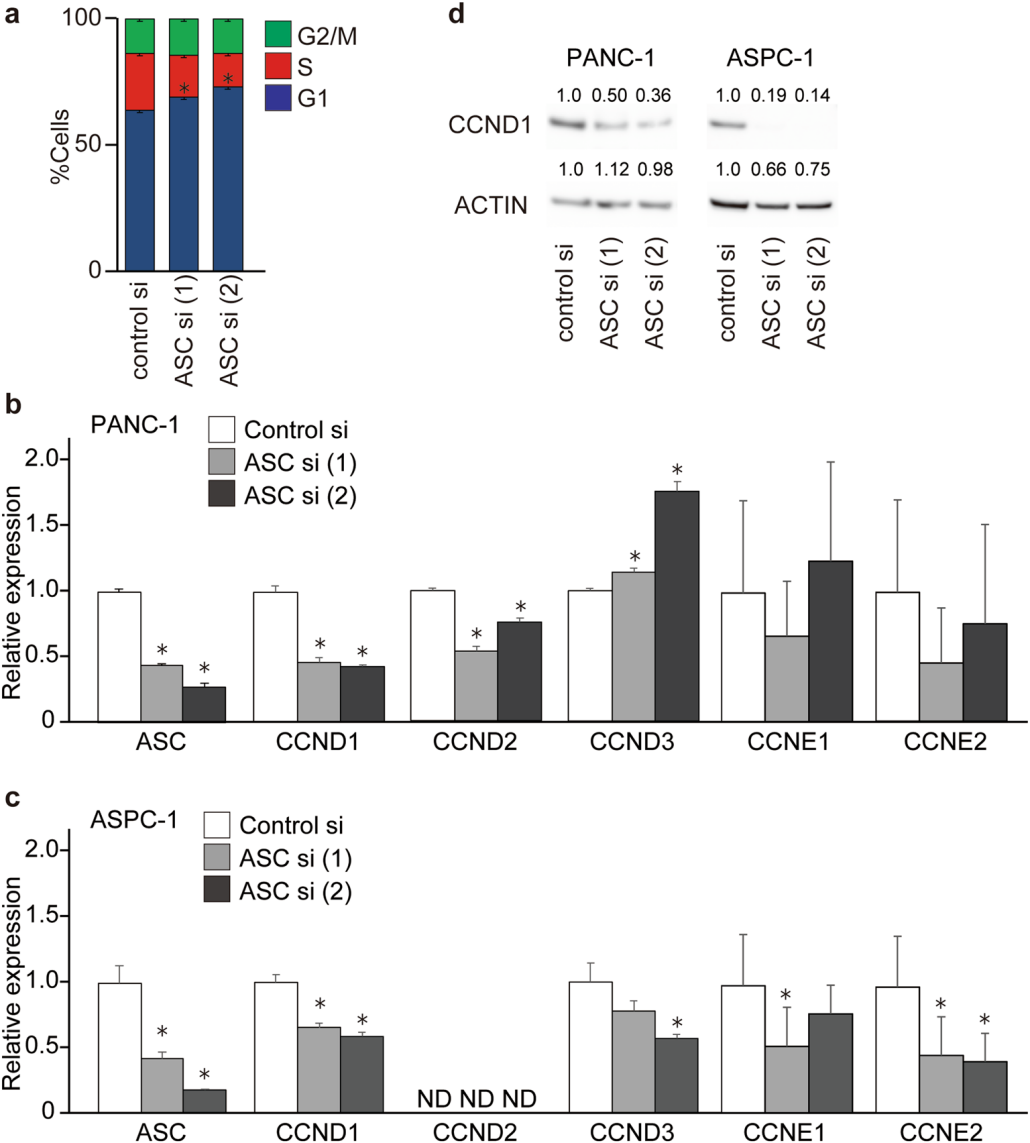
*ASC* silencing reduces CCND1 expression and blocks the cell cycle at the G1 phase. (**a**) Quantification of three experimental replicates of cell cycle phase distribution. Error bars indicate the standard error of the mean based on three independent experiments. *p < 0.05 vs. control, Student’s *t* test. (**b**, **c**) mRNA levels of *CCND*s and *CCNEs* according to real-time RT-PCR following *ASC* silencing in PANC-1 and ASPC-1 cells. Data represent the mean ± standard error of the mean of three to four replicates. (**d**) CCND1 expression following *ASC* silencing according to western blot. Relative densitometry data as compared with controls are given for each lane. The original gel images of Western blotting are shown in Fig. S8.

We then employed flow cytometry to determine whether ASC knockdown indeed affected the cell cycle in PDAC cells, with the results showing a substantial increase in the population of cells in the G1 phase and a concomitant reduction of cells in the S phase (Fig. 6a). Based on this result, we focused on CCNs that regulate the cell cycle in the G1 phase. Real-time RT-PCR revealed that expression of the G1-phase gene *CCND1* was decreased by *ASC* silencing in PANC-1 cells and AsSPC-1 cells (Fig. 6b,c), whereas *CCND2* expression was decreased in PANC-1 and undetected in AsPC-1 cells. Additionally, *CCND3* expression increased in PANC-1 cells and decreased in AsPC-1 cells, and we observed no change in *CCNE1* and *CCNE2* expression in PANC-1 cells but slight reductions in AsPC-1 cells. Western blotting subsequently confirmed changes in CCND1 protein levels corresponding to the observed downregulation of gene expression in both cell lines (Fig. 6d). These results suggested that *ASC*-silenced cells were arrested in the G1 phase due to regulated expression of cell cycle-related genes, which subsequently decreased cell proliferation.

### Downregulation of ASC level does not modulate the expression of apoptosis-related molecules in PDAC cells

**Figure 7:**
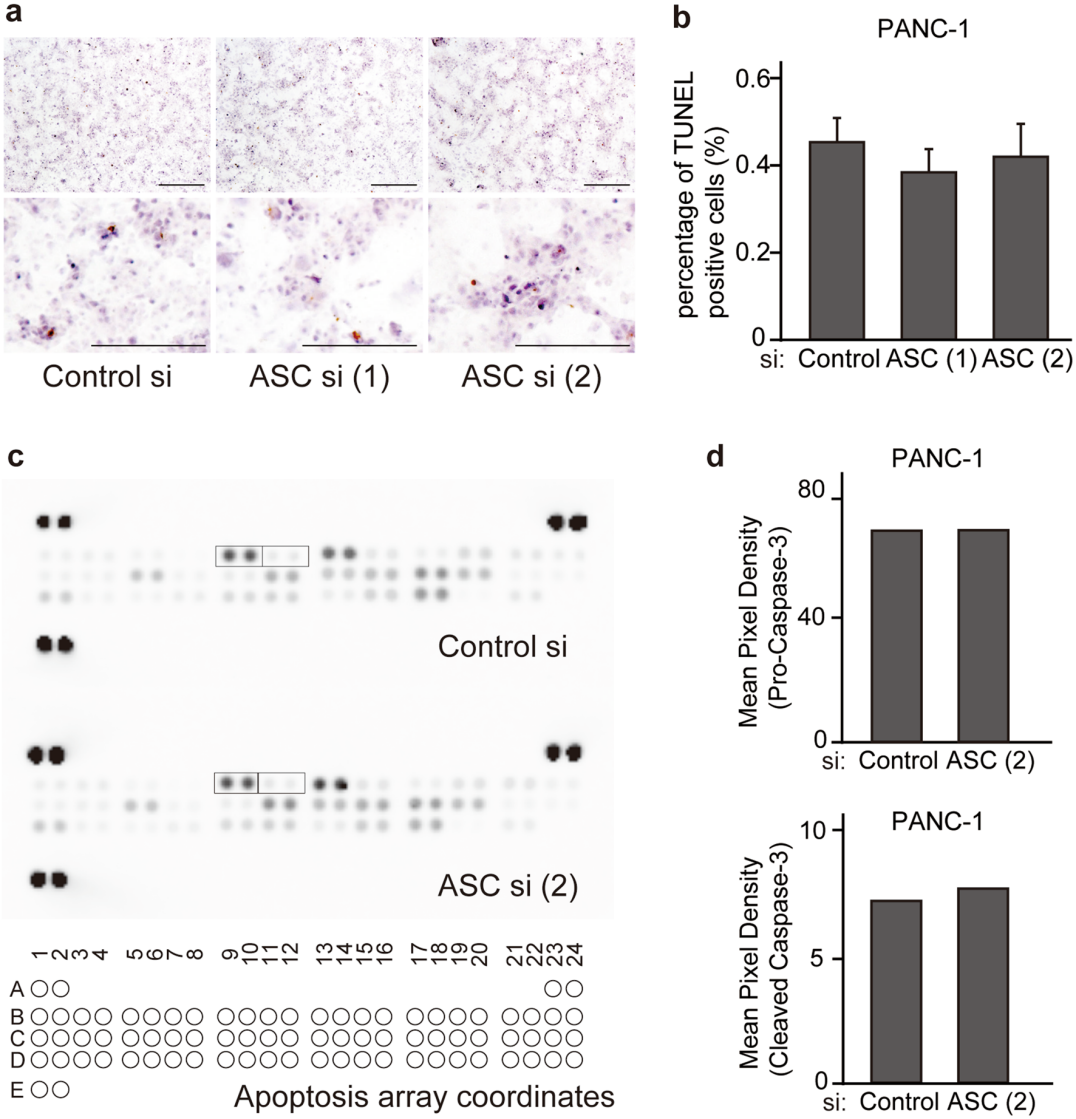
*ASC* silencing does not induce apoptosis-related changes in PDAC cells. (**a**) IHC staining of PANC-1 cells to detect apoptosis induction and TUNEL staining at 72-h post-transfection with control and *ASC* siRNA. Scale bar: 500 µm. (**b**) Quantitation of apoptotic cells based on TUNEL staining at 72-h post-transfection with control and ASC siRNA. Data represent the mean ± standard error of the mean of five areas. (**c**) Changes in the expression of apoptosis-related proteins at 72-h post-transfection with control and *ASC* (2) siRNA in PANC-1 cells and analyzed using human apoptosis proteome profiler arrays. Boxes show the localization of procaspase-3 and cleaved caspase-3 on the membrane. The cytokine array coordinates are as follows: positive ctrl (A1, A2); positive ctrl (A23, A24); Bad (B1, B2); Bax (B3, B4); Bcl-2 (B5, B6); Bcl-x (B7, B8); procaspase-3 (B9, B10); cleaved caspase-3 (B11, B12); catalase (B13, B14); cIAP-1 (B15, B16); cIAP-2 (B17, B18); claspin (B19, B20); clusterin (B21, B22); cytochrome c (B23, B24); TRAIL R1/DR4 (C1, C2); TRAIL R2/DR5 (C3, C4); FADD (C5, C6); Fas/TNFRSF6/CD95 (C7, C8); HIF-1α (C9, C10); HO-1/HMOX/HSP32 (C11, C12); HO-2/HMOX2 (C13, C14); HSP27 (C15, C16); HSP60 (C17, C18); HSP70 (C19, C20); HTRA2/0mi (C21, C22); livin (C23, C24); PON2 (D1, D2); p21/IP1/CDKN1A (D3, D4); p27/Kip (C5, D6); phospho-p53 (S15) (D7, D8); phospho-p53 (S46) (D9, D10); phospho-p53 (S392) (D11, D12); phospho-Rad17 (S635) (D13, D14); SMAD/Diablo (D15, D16); survivin (D17, D18); TNF RI/TNFRSF1A (D19, D20); XIAP (D21, D22); negative ctrl (D23, D24); and positive ctrl (E1, E2). (**d**) Quantitative analysis of procaspase-3 and cleaved caspase-3. Data represent the average of two spots.

ASC is reportedly associated with apoptosis; therefore, we performed TUNEL staining and antibody arrays to observe changes in apoptosis in PDAC cells due to ASC downregulation. TUNEL staining revealed no changes in apoptosis upon *ASC* silencing at 3- and 5-days post-transfection with control and *ASC* siRNA (Figs. 7a,b; and S4). Furthermore, examination of apoptosis using antibody arrays revealed no changes in the expression of apoptosis-related proteins, including caspase-3, upon ASC downregulation (Fig. 7c,d).

## Discussion

In recent years, increasing evidence has shown that ASC plays an important role in the interaction between PDAC cells and cells in the surrounding microenvironment (e.g., tumor-associated macrophages and cancer-associated fibroblasts)^27,28^; however, the role of ASC in signal transduction in PDAC cells has not been fully investigated. The present study demonstrated that downregulation of *ASC* induced changes in cell cycle in PDAC cells and inhibited proliferation.

A previous report identified methylated regions associated with *ASC* in 23% of papillary thyroid carcinomas and none in normal thyroid samples^29^. In other carcinomas, *ASC* methylation has been reported in 63.6% of prostate cancer, 40% of breast cancer cases, 32.1% of lung adenocarcinomas, 13.2% of lung squamous cell carcinomas, 38.5% of lung large-cell carcinomas, and 60% of lung small-cell carcinomas^10,29,30^. Although there are no studies on the frequency of *ASC* methylation in PDAC, a previous report indicates that > 90% of PDAC cells are IHC-positive for ASC^28^. In the present study, we identified ASC overexpression in human PDAC cells through bioinformatics methods. Additionally, IHC confirmed that ASC was more highly expressed in tumor tissues than in paired paracancerous tissues, as previously reported^28^. ASC expression is enhanced in ducts near the tumor and in metaplastic ducts and further enhanced in cancer, although rarely seen in ducts distant from the tumor. This suggests that ASC may be involved in acinar-duct metaplasia, which contributes to the development of pancreatic cancer, and might be an important factor for carcinogenesis.

Moreover, three of four PDAC cell lines exhibited ASC overexpression relative to levels observed in hTERT-HPNE cells generated from human pancreatic duct samples with acinar cells associated with the ductal metaplasia phenotype^31^. These results indicated that *ASC* expression might be suppressed by methylation in PDAC cell lines, such as MIA PACA-2; however, ASC is also considered to be highly expressed relative to levels observed in non-cancerous tissue. These results suggest that *ASC* methylation and expression vary according to the tissue and cancer type. Protti et al.^15^ consider ASC as a tumor suppressor when its expression in tumor cells is lower than that in normal epithelial cells and non-tumor adjacent tissues and tumor-promoting when its expression in tumor cells is higher than that in normal tissue. In the present study, we found that elevated *ASC* mRNA expression in tumor tissues was a factor associated with poor prognosis for PDAC. Tumors with low mutational burden are reportedly less responsive to current immunotherapies and require new therapeutic targets. Therefore, ASC might represent a potential therapeutic target in tumors with low mutational burden, as we found that *ASC* expression is a prognostic factor for PDAC. In particular, it would by clinically important as a prognostic factor in grade 2 tumors, which are the most common in PDAC^32^. These findings suggest that ASC might be an essential biomarker of PDAC and contribute to PDAC progression.

There has been one report on ASC using PDAC cells. The results of this report showed that increased *ASC* expression due to demethylation in MIA PaCa-2 cells resulted in increased sensitivity to gemcitabine and docetaxel^33^; however, there have been no studies reporting the detailed molecular function(s) of ASC in PDAC. Previous reports indicate that ASC plays an important role in apoptosis induction in various cancers^34,35^; however, in the present study, we observed no induction of apoptosis following *ASC* silencing in PDAC cells. Additionally, in IMR90-E1A cells, *ASC* silencing significantly inhibited etoposide-induced apoptosis; however, in the absence of etoposide stimulation, there was no change in the percentage of apoptotic cells relative to controls^36^. These findings suggest that ASC does not affect apoptosis in pancreatic cancer cells, at least in the absence of exogenous apoptotic stimuli. On the other hand, ASC is also a key adaptor molecule of inflammasomes and activates procaspase-1, which is necessary for processing IL-1β and IL18^37,38^. In the present study, we found that IL-1β level in the supernatant was below the measurement sensitivity in PANC-1 and AsPC-1 cells (Fig. S5). Moreover, RNA-seq showed that *ASC* silencing did not induce changes in inflammasome-related gene groups (data not shown). Therefore, these findings suggest that ASC does not contribute to changes in apoptosis or inflammasome formation in PDAC cells.

We observed suppression of PDAC cell proliferation following *ASC* silencing; however, this was not observed in MIA PACA-2 cells with poor ASC expression, suggesting that this is not an off-target effect. Additionally, because ASC-related effects were not observed in hTERT-HPNE cells, this suggests that ASC-specific effects are unique to PDAC cells. A previous report demonstrated that *ASC* silencing resulted in reduced cell viability, suppressed tumor growth, and arrested the cell cycle in the G1 phase in metastatic melanoma. The authors hypothesized that ASC might contribute to a positive feedback loop in cases of autoinflammation, where upregulated nuclear factor-κB expression results in transactivation of pro-IL-1β to promote autocrine IL-1 signaling in metastatic melanoma^13^. However, because no IL-1β was detected in the culture supernatant of PDAC cells in the present study and a previous report^39^, we speculated that other pathways are responsible for the changes in cell proliferation. Here, RNA-seq analysis showed that *ASC* silencing induced changes in the expression of cell cycle-related genes. The G0/G1 phase of the cell cycle is regulated by CCNs, including CCND and CCNE, among which we observed significant changes in only *CCND1* expression. *CCND1* and *CCND3* are often differentially overexpressed in PDAC^40^, and CCND2 reportedly plays a role in the proliferation of pancreatic islet β-cells^41^, although its expression is infrequently detected in PDAC tissues and cells^40,42^. In the present study, *ASC* silencing induced a significant reduction in *CCND1* expression in PANC-1 and AsPC-1 cells, whereas *CCND3* expression increased in PANC-1 cells and decreased in AsPC-1 cells, and *CCND2* was undetectable in AsPC-1 cells. Moreover, *CCNE1* and *CCNE2* expression did not show the same changes in PANC-1 and ASPC-1 cells. Based on these results, we speculated that CCND1 contributes to the change in cell proliferation observed following *ASC* silencing in PDAC cells. CCND1 expression is associated with anchorage-independent growth, tumorigenicity, angiogenesis, hypoxia response, and resistance to chemotherapeutic agents^43^. Furthermore, it plays an important role in G1/S-phase transition and cell proliferation^44^. Because *ASC* silencing resulted in an increase in the population of cells in the G1 phase and a concomitant reduction of cells in the S phase, we suggest that ASC regulates *CCND1* expression and promotes G1/S-phase transition.

In summary, we found that downregulated ASC expression inhibited tumor progression by modulating the cell cycle in PDAC cells. These findings suggest ASC as a possible therapeutic target in PDAC; however, further studies are warranted to investigate this possibility.

## Supplementary Information

Supplementary Information.

## Data Availability

The datasets generated and/or analyzed during this study are available from the corresponding author upon reasonable request.
