# Supplementary material for: Apoptosis-associated speck-like protein containing a CARD regulates the growth of pancreatic ductal adenocarcinoma
Source: Sci Rep. 2021 Nov 16;11:22351. doi: 10.1038/s41598-021-01465-2 (PMC8595714; doi:10.1038/s41598-021-01465-2)
Supplement: Supplementary file 1 — Supplementary Information. [file 41598_2021_1465_MOESM1_ESM.docx]

**Supplementary Information**

**Apoptosis-associated speck-like protein containing a CARD regulates the growth of pancreatic ductal adenocarcinoma**

Mitsuhito Koizumi, Takao Watanabe, Junya Masumoto, Kotaro Sunago, Yoshiki Imamura, Kozue Kanemitsu, Teru Kumagi, Yoichi Hiasa

Supplementary Figure S1. *ASC* mRNA expression in different PDAC cell lines according to the CCLE.


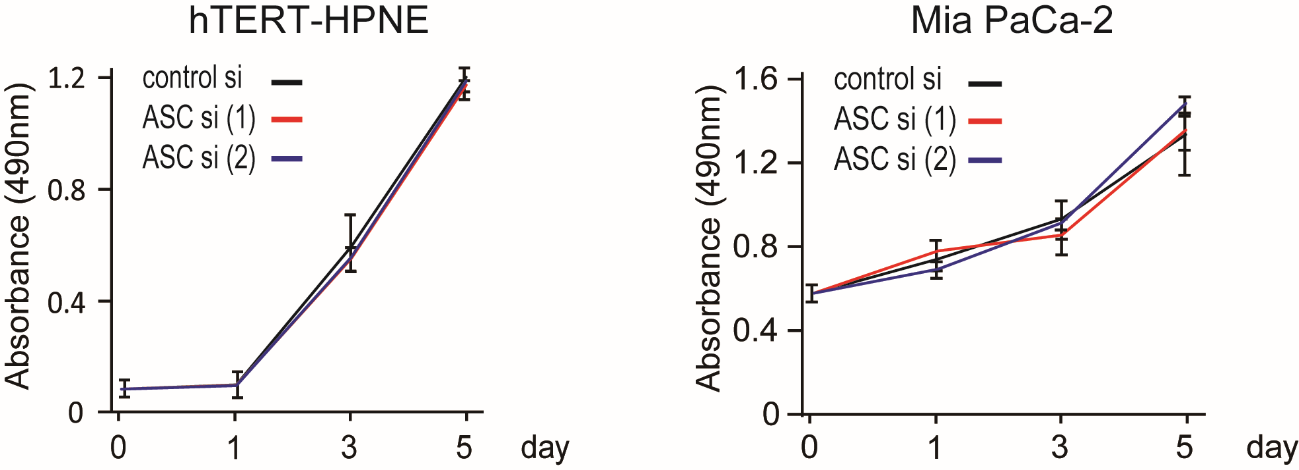


Supplementary Figure S2. Viability of cells transfected with control or *ASC* siRNA. Data represent the mean ± standard error of the mean of three replicates.


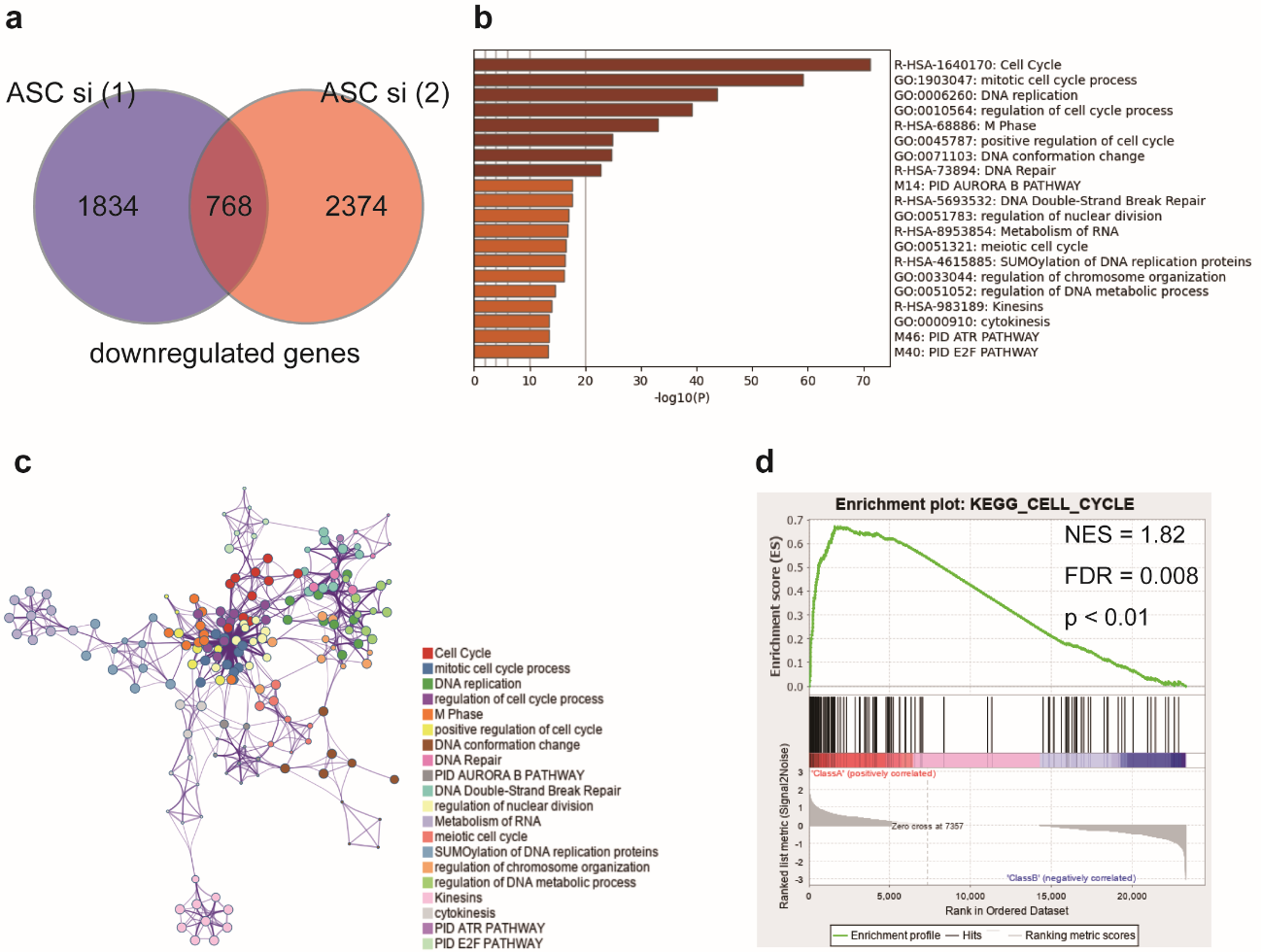


Supplementary Figure S3. Venn diagram showing overlapping RNAs detected via RNA-seq analysis of downregulated RNAs at 72-h post-transfection of either *ASC* (1) or (2) siRNA and relative to control siRNA-transfected AsPC-1 cells. (b) Functional enrichment results. Heatmap showing the top 20 clusters colored according to p-value (a darker color indicates a lower p-value). (c) Network of the top 20 clusters of enriched terms. Each node indicates an enrichment term colored by cluster ID. Nodes sharing the same cluster are generally located in close proximity. Terms with a kappa similarity ≥0.3 are connected. (d) GSEA for the gene signature of KEGG_CELL_CYCLE.

NES, normalized enrichment score; FDR, false discovery rate.


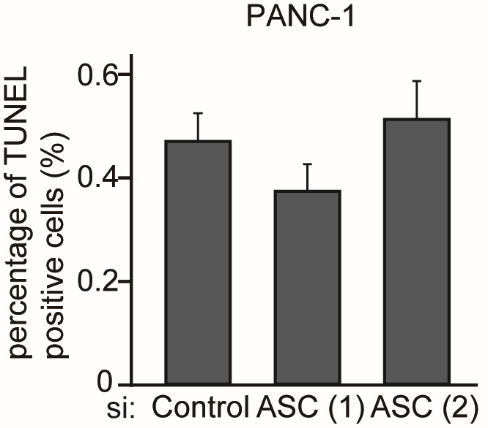


Supplementary Figure S4. Quantitation of apoptotic cells based on TUNEL staining at 5-days post-transfection with control and *ASC* siRNA.


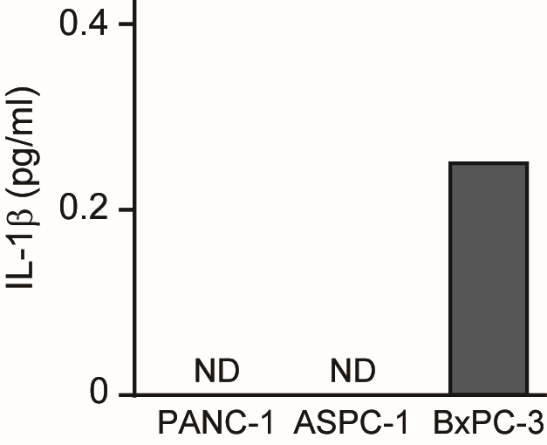


Supplementary Figure S5. IL-1β secretion by PDAC cells. IL-1β levels in the supernatant of PDAC cells were detected using ELISA.


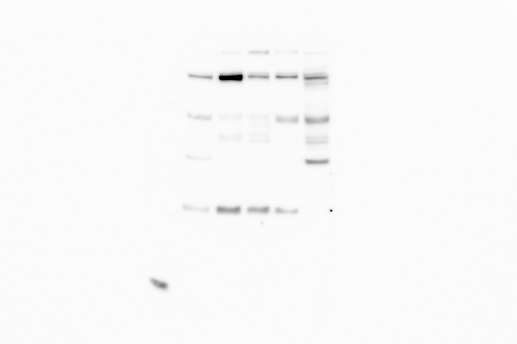

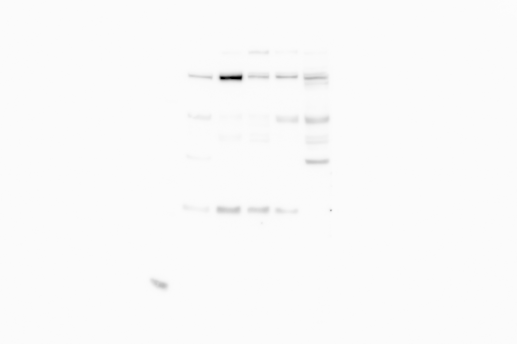


ASC

22kDa

50 sec

100 sec


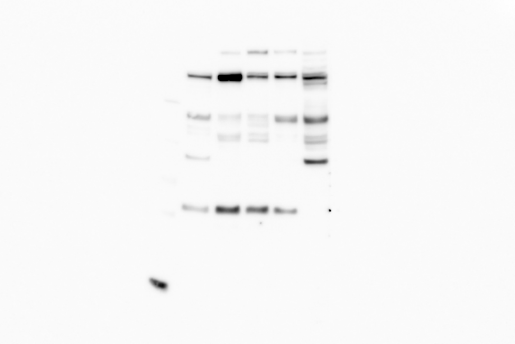

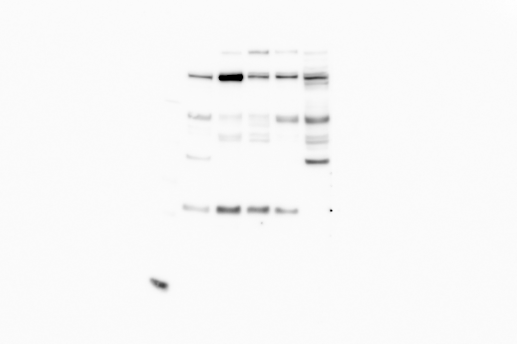


ASC

22kDa

200 sec

150 sec


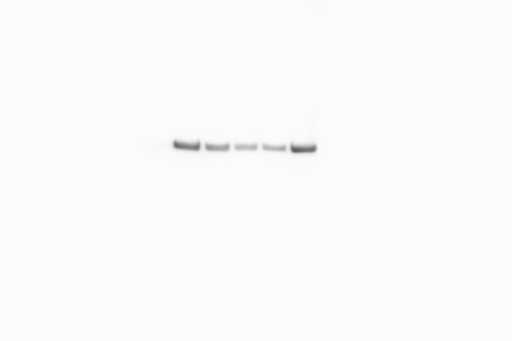

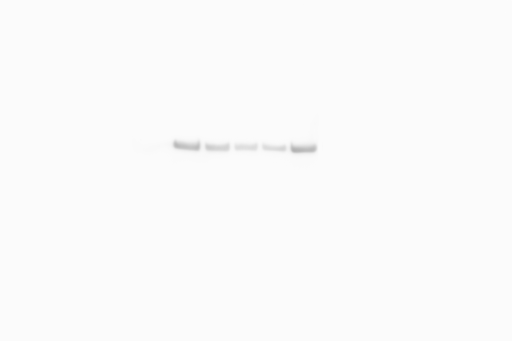


ACTIN

42kDa→

5 sec

10 sec


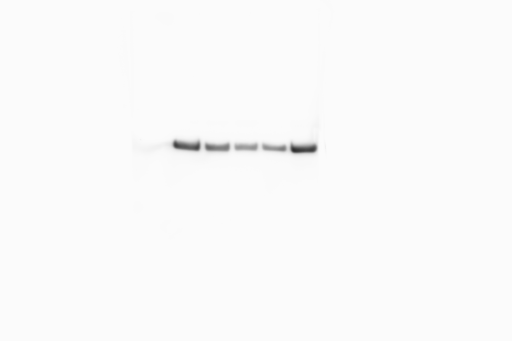

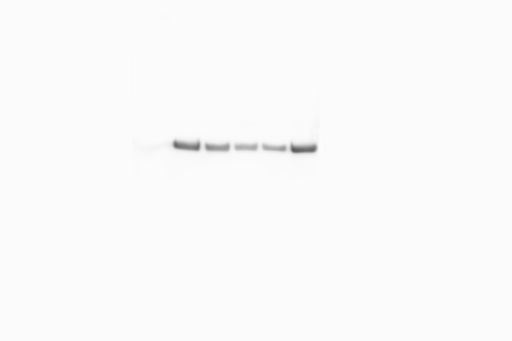


ACTIN

42kDa

15 sec

20 sec

Supplementary Figure S6. Original blots for Figure 3b. Blots with exposure times shown in the lower left square were described.


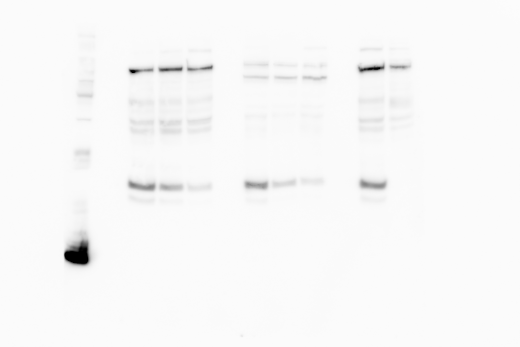

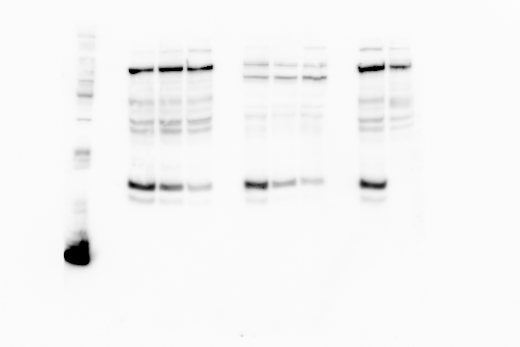


ASC

22kDa

100 sec

50 sec


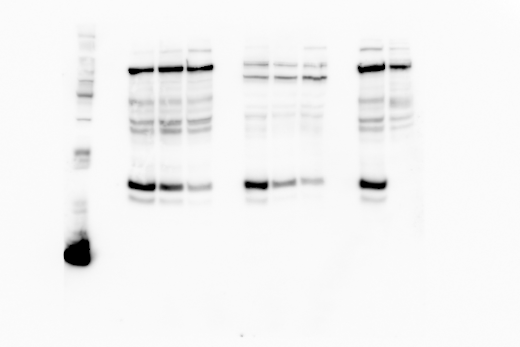


ASC

22kDa

150 sec

AsPC-1

PANC-1


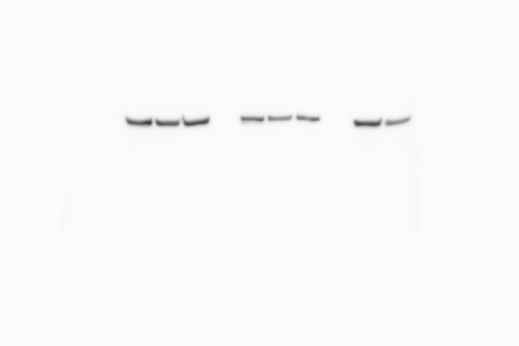

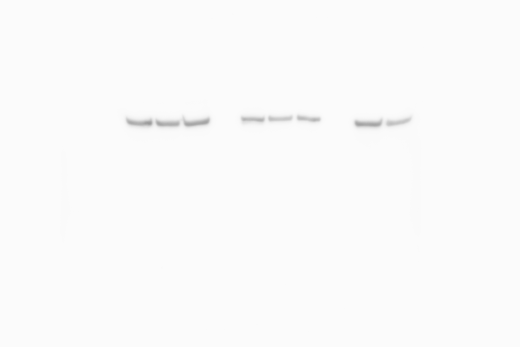


ACTIN

30 sec

42kDa

15 sec

AsPC-1

PANC-1

PANC-1

AsPC-1

Supplementary Figure S7. Original blots for Figure 4b. Blots with exposure times shown in the lower left square were described.


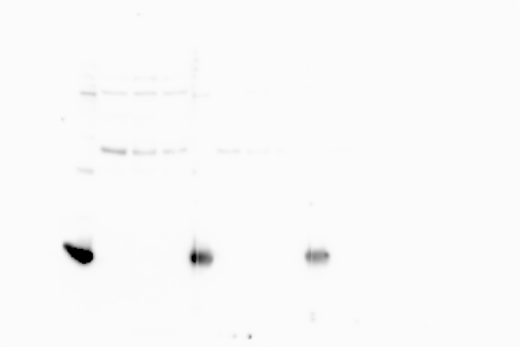

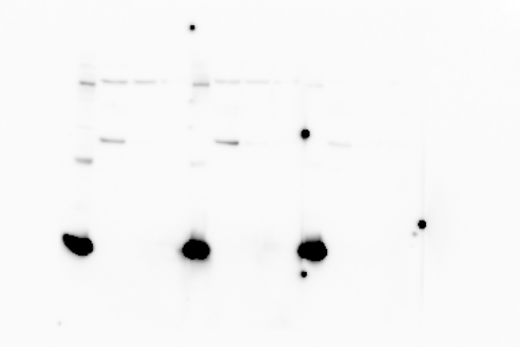


CCND1

34kDa

70 sec

30 sec


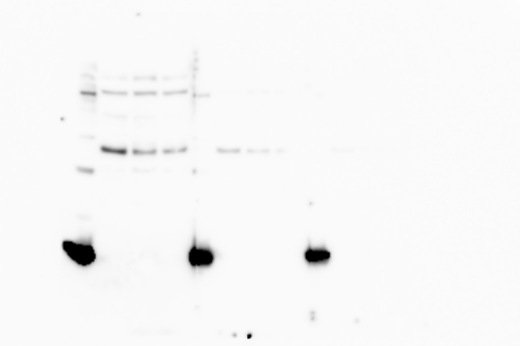

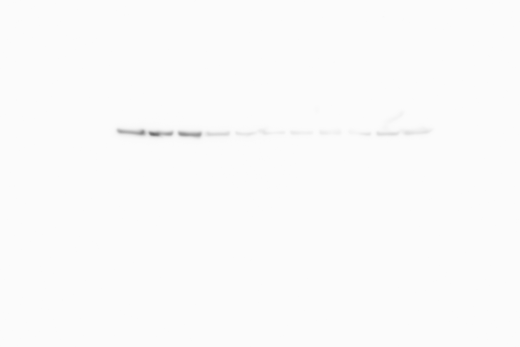

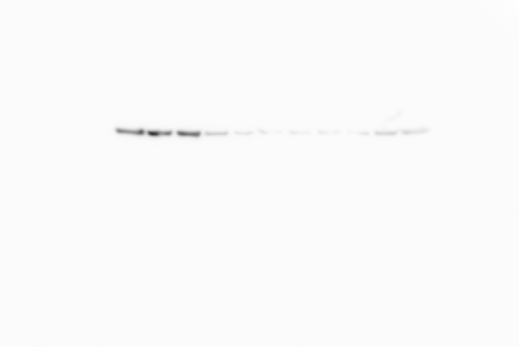

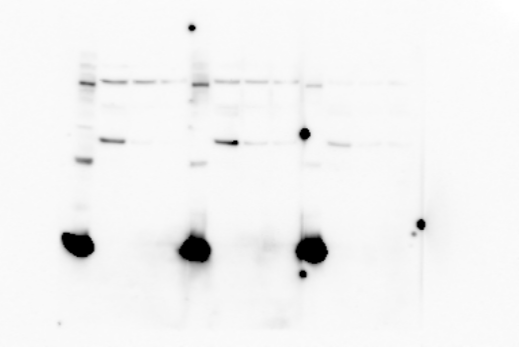


CCND1

34kDa

90 sec

140 sec

PANC-1

AsPC-1


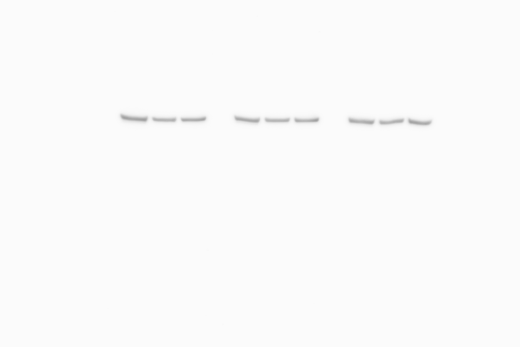


ACTIN

42kDa

10 sec

30 sec


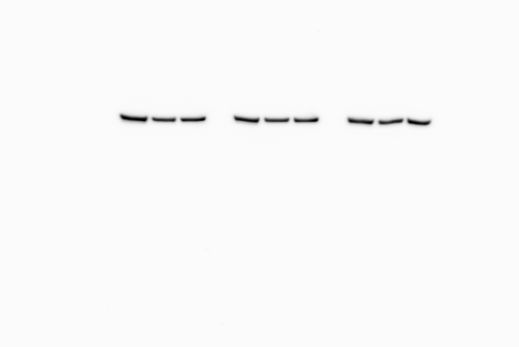


ACTIN

42kDa

90 sec

70 sec

PANC-1

AsPC-1

Supplementary Figure S8. Original blots for Figure 6d. Blots with exposure times shown in the lower left square were described.

**Table S1. Primer sequences used for real-time PCR.**

| *ASC* | Forward primer | GCCAGGCCTGCACTTTATAGA |
| --- | --- | --- |
|  | Reverse primer | GTTTGTGACCCTCGCGATAAG |
| *CCND1* | Forward primer | GAAGATCGTCGCCACCTG |
|  | Reverse primer | GACCTCCTCCTCGCACTTCT |
| *CCND2* | Forward primer | GGACATCCAACCCTACATGC |
|  | Reverse primer | CGCACTTCTGTTCCTCACAG |
| *CCND3* | Forward primer | ATCACTGGCACTGAAGTGGA |
|  | Reverse primer | CCTGAGGCTCTCCCTGAGT |
| *CCNE1* | Forward primer | GCAGGATCCAGATGAAGAAATG |
|  | Reverse primer | TATTGTCCCAAGGCTGGCTC |
| *CCNE2* | Forward primer | AGAGGAAAACTACCCAGGATGTC |
|  | Reverse primer | AATGCAAGGACTGATCCCCC |
| *GAPDH* | Forward primer | AGCCACATCGCTCAGACAC |
|  | Reverse primer | GCCCAATACGACCAAATCC |

**Table S2.** Antibodies used in this study.

| Antibody | Company | Protocol |
| --- | --- | --- |
| ASC | Proteintech, 10500-1-AP | IHC, WB |
| CCND1 | Proteintech, 26939-1-AP | WB |
| β-actin | Millipore, MAB1501 | WB |

IHC, immunohistochemistry. WB, western blot.
